# Load-Oriented Nonplanar Additive Manufacturing Method for Optimized Continuous Carbon Fiber Parts

**DOI:** 10.3390/ma16030998

**Published:** 2023-01-21

**Authors:** Johann Kipping, Thorsten Schüppstuhl

**Affiliations:** Institute for Airplane Production Technology, Hamburg University of Technology, 21073 Hamburg, Germany

**Keywords:** carbon-fiber-reinforced polymers, nonplanar slicing, additive manufacturing, multi-axis motion, 3D printing, path planning, continuous fiber composites

## Abstract

The process of the additive manufacturing (AM) of carbon-fiber-reinforced polymer (CFRP) parts based on the process of fused deposition modeling (FDM) has seen considerable research in recent years, which amplifies the importance of adapted slicing and pathplanning methods. In particular, load-oriented techniques are of high interest when employing carbon fiber materials, as classical methods, such as tape-laying and laminating, struggle with highly curved and complex geometries and require the costly production of molds. While there have been some promising propositions in this field, most have restricted themselves to a planar slicing approach, which severely limits the ability to place the fibers along stress paths. In this paper, a nonplanar slicing approach is presented that utilizes principal stress directions to construct optimized nonplanar constituting layers on which pathplanning can be carried out. These layers are oriented such that the effect of the weak interlayer adhesion is minimized. Support material is adaptively generated to enable the use of arbitrary part geometry. Furthermore, a continuous pathplanning method and post-processor are applied to yield manufacturing instructions. The approach is verified for its viability of application through experimental investigation on a multi-axis robotic 3D printer. This constitutes an important step in allowing the fabrication of CFRP parts to further utilize the possibilities of additive manufacturing.

## 1. Introduction

Additive manufacturing (AM) is the process of building up material in layers. Especially in recent years, an increase in researchers’ attention could be observed, as complex parts can quickly be manufactured with high efficiency [1,2]. The usage of computer-aided design (CAD) software allows a high degree of freedom in design. Fused deposition modeling (FDM), as a subcategory of AM, is the process of constructing a part by melting and extruding a material through a nozzle, fusing extruded filament to the existing structure. Carbon-fiber-reinforced polymers (CFRPs) are composite materials achieving high strength-to-weight ratios, with motivates their increasing application in domains such as aerospace, automotive and many other areas, where weight and strength are key factors [3]. These properties stem from the high stiffness of the carbon fiber in combination with the polymer matrix, which provides a continuous surface and distributes the load among the fiber strands, which exhibit high degrees of anisotropy by themselves. Combining FDM and CFRP in an attempt to combine the flexibility and efficiency of AM with the performance of carbon fiber composites has been regarded as having high potential, leading to active research in this field over the last few years [3,4]. An early approach was the use of filaments containing chopped fibers. Although a pronounced increase in mechanical performance can be observed, the high amount of discontinuities prevent this material from being able to compete with classical composites [5,6]. In the case of continuous fibers, the load along the filament’s length is transferred not through the matrix, but through the fiber itself, greatly improving the mechanical performance. The potential of AM compared to classical manufacturing methods, such as filament winding, automated fiber placement and automated tape laying, lies in the shorter development cycles, lower material waste and especially in the possibilities of geometric complexity. Zhuo et al. state in [6] that this perspective is only feasible if fiber directions are designed and optimized to make the most out of the possibility to guide the fiber path. Following Luca et al. from [7], the need for optimized slicing and pathplanning software is greater than ever for AM if CFRP is to realize its full potential.

The method presented in this paper aims to deliver adapted manufacturing instructions for multi-axis 3D printers, utilizing the ability to place fibers out-of-plane to produce nonplanar layers that follow the principal stress directions. This is achieved by obtaining the constituting layers as isosurfaces of an optimized scalar field, which is computed from the vector field of minimum principal directions. This ensures that the weaker interlayer adhesion has minimal effect on the manufactured part. After generating support material, a continuous pathplanning method based on the first-in-spiral-out (FISO) algorithm is applied to remove the necessity of cutting the filament while fabricating a layer. Experimental evaluation with a demo part was performed to confirm the viability of the approach. The structure of the paper is as follows: In the next sections, prior works are discussed and the developed procedure is presented. Section 2 gives an overview and explanation of the employed techniques, and the method is formally derived. In Section 3, the approach is described and explained in detail. Section 4 and Section 5 contain the results of the experimental verification of viability and the conclusive statement.

### 1.1. Prior Work

As pathplanning is of high importance for a successful implementation of the manufacture of CFRP parts using FDM, there has been a wide variety of publications in recent years, which will be presented in the following. Starting with planar algorithms, the main focus will be on the incorporation of load-orientation and topology optimization. After this, the presentation of nonplanar algorithms will follow. The focus hereby lies on volumetric bodies, mostly excluding thin-walled structures and sandwich panels. The applied nomenclature has be adapted from [8].

Slicing the part with parallel planes represents the classical approach to generating constitutive layers on which pathplanning can be performed. Marchal et al. investigated the influence of planar pathplanning and the effectiveness of the rudimentary strategies currently offered by industrial applications in [9]. After experimental evaluation, they came to the conclusion that a load-oriented approach is essential for good mechanical performance, which is currently not offered by any commercial software. Based on this observation, which has been validated by several other publications [10,11,12], the following will concentrate on load-oriented approaches. The most popular method in this regard is the utilization of finite-element-analysis (FEA) with carefully selected boundary conditions to obtain a stress distribution. After applying an principal axis transform to the corresponding stress tensor, the principal stresses and principal axes can be obtained as the tensor’s eigenvalues and eigenvectors, respectively. The principle of reinforcement [13] states that having the directions of the anisotropic fibers align with the maximum principal axis at every point can provide profound mechanical improvement for a part under given loads. One of the main difficulties with this approach is the nontrivial structure of the vector field of principal directions. High turbulence and divergence lead to the inability to perfectly align the fiber direction with this field. Chen et al. present a promising approach in [10], where this problem is tackled by first computing an optimal vector field to increase the compatibility of neighboring vectors. In a next step, they find a scalar field by minimizing its gradient’s difference to the vector field. Applying the level-set method yields isocurves, which can be used as trajectories for fiber deposition. Additionally, the fiber density is adaptively set, proportional to the local maximum principal stress. The greatest drawback of this is the inherently low maximum possible fiber content. One of the earliest publications following a similar approach was [14] by Yamanaka et al., directly employing the streamlines of perfect flow as fiber paths. This ensured continuity and the absence of intersection, yet also suffered from low fiber density. In [15], Sugiyama et al. put the focus on varying the fiber volume fraction through pathplanning and experimentally validated an increase in stiffness and strength. Similarly, Sun et al. used streamlines in conjunction with a greedy algorithm and downsampling, yielding low fiber density as well. Another solution for reducing the vector field complexity is prior topology optimization of the part with adapted pathplanning. In this spirit, Wang et al. applied solid orthotropic material with penalization (SOMP), a method based on solid isotropic material with penalization (SIMP), restricting turbulence to junctions almost exclusively and reducing divergence. The medial axis was computed using a voronoi diagram, which serves as the base for pathplanning in conjunction with the assumption of minimal divergence [16]. Using topology optimized parts further utilizes the process of additive manufacturing, as classical manufacturing methods struggle to realize the resulting complex geometries. In a similar approach, Li et al. [17] laid the groundwork by identifying and separating scaffold beams and branches as well as areas of tensile and compressive stress and experimentally verified the approach.

One problem with the previously discussed approaches is outlined by Qiu et al. in [18]. Employing multiple load cases can lead to the inability of principal axes to adequately represent the optimal fiber directions. Following [19], this can be disregarded in most cases when shear weak materials are used. An alternative to the approach of using principal directions is presented by Avanzini et al. [20]. They applied the embedded elements method, the effectiveness of which could not be sufficiently verified.

There exist promising approaches that combine topology optimization and pathplanning for CFRP parts by simultaneously computing material density and fiber direction. These will be presented in the following. In the publication [21] by Schmidt et al., a general approach for working with anisotropic materials on the basis of SIMP is presented. The algorithm achieves a groundbreaking level of generality, as it is applicable in 2D and 3D cases, while being agnostic to material properties, such as stiffness, conductivity and other physical properties. Furthermore, care is taken to ensure numerical stability and enable the use of multiple load cases. A multiscale method is presented by Huang et al. in [22], where the paths follow the directions computed during topology optimization. In the macroscopic realm, a Hamilton path is computed over a partition of the part to obtain the routing.

The field of nonplanar pathplanning for CFRP parts is in its early days. The few proposed strategies have limited applicability and often require manual work. Therefore, the following will briefly discuss these methods to then expand to nonplanar slicing and pathplanning for classical FDM. Zhang et al. were one of the first groups to investigate the process of nonplanar manufacturing of CFRP with FDM in [23]. They verified the process and further stated the necessity for adapted software solutions. Major improvement in mechanical performance was observed for the simple nonplanar specimen compared to the planar control specimen. Another publication stems from the architectural domain. Kwon et al. applied the robotic AM of CFRP in [24] to the field of producing architectural panels. Carbon fibers were placed along lines of tensile stress on the freeform outer surfaces. To be able to apply planar pathplanning using fermat-spirals to nonplanar parts, Yao et al. limited themselves to developable surfaces in [25]. This limitation was overcome in [26], where several methods for pathplanning in nonplanar surfaces are proposed. The slicing of a part, meaning computation of the surfaces itself, is omitted in this publication, and user input is still required for most of the developed algorithms. As this concludes the nonplanar approaches for CFRP, the scope of this literature is extended to the itself young field of nonplanar slicing and pathplanning for FDM. For a full list of proposed algorithms, consult Table 2 in [8], as some of simpler approaches are omitted here. In general, the most promising modern approaches fall into two categories: field-based and decomposition-based. Li et al. present a new method in [27] using a geodesic field to extract isosurfaces, which guarantee uniform layer thickness. This intrinsic approach has great potential for nonplanar printing due to its generality, although it has been very limited in its ability to consider load analysis. With the publication [28], Wulle et al. highlight the importance of pathplanning and denote the possibilities for both support-free and load-oriented printing as the main advantages of nonplanar printing. In a follow-up paper [29], they refine the previous load-oriented approach based on decomposition. A breakthrough in load-oriented slicing and pathplanning is introduced by Fang et al. in [13], which serves as the inspiration and starting point for the work presented in this paper. They adopt the approach of computing isosurfaces of an optimized scalar field based on the vector field of principal directions from a FEA to ensure that the nonplanar constituting surfaces comply with the load case.

In conclusion, it can be said that the presented approaches demonstrate both the importance and the difficulties of a load-oriented slicing and pathplanning solution for the manufacture of CFRP parts with FDM. The method presented in the following represents an important step in enabling progress in this field.

### 1.2. Developed Method

In order to derive an adequate method for the load-oriented slicing and pathplanning of volumetric parts, a deduction of the approach presented in this paper was conducted based on the criteria and requirements, which are described in detail in Section 2. A central element in the computation of slices is the observation that the interlayer adhesion can be regarded as the weakest link in a volumentric additively manufactured CFRP part, similarly to the layup process and classical FDM. This leads to the conclusion that the surface normals should coincide with the minimum principal direction to minimize this effect. On this basis, the following framework is proposed:

1. The procedure starts by conducting an FEA in an external software to extract the major principal stresses and direction vectors. Boundary conditions are defined and the geometry is established, possibly through topology optimization. The output of this process is a tetrahedral mesh with information on the stress tensor stored for every element.
2. To circumvent the sometimes highly turbulent and divergent properties of the field of minimal principal directions, critical regions in the part are identified. These are determined as the set of elements that surpass a user-defined threshold and then are labeled corresponding to the region’s connectivity and compatibility.
3. The minimum principal directions in these regions are used as the source for a construction of an optimized vector field. This is performed by minimizing the Dirichlet energy, resulting in smooth transitions between the regions. Special attention has to be paid to the ambiguity of orientation, as the coordinate frames resulting from the principal axis transform are independent of sign.
4. The optimized scalar field is then computed by minimizing the difference of its gradient to the determined vector field.
5. After computing the supporting structures geometry, the process above is repeated with a critical region being added at the printing bed to ensure the first layer being planar. Slicing the scalar field at fixed values corresponding to the user-defined layer height yields the constituting surfaces for both the support and part. This ensures the compatibility of the layers along the parts outer surface.
6. Pathplanning with the continuous FISO algorithm is executed, and the subpaths are connected with travel motions.
7. Finally, post-processing of the path results in machine instructions to be executed on a multi-axis printer.

**Figure 1:**
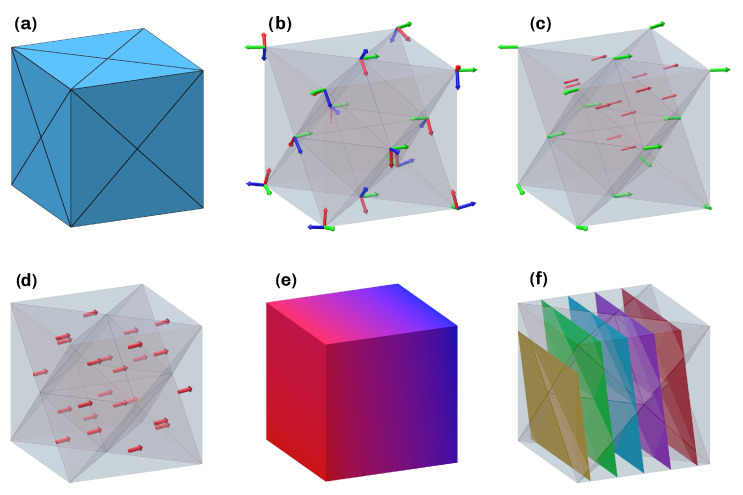
Simplified overview of the approach. (**a**) Tetrahedral mesh of a cube. (**b**) Principal direction at the vertices. (**c**) Minimum principal directions (green), optimized vector field in critical regions (red). (**d**) Extrapolated vector field on the cells. (**e**) Scalar field. (**f**) Constituting surfaces.

A simplified overview of the steps 1–4 is shown in Figure 1. This paper aims to formally describe the derivation and implementation of the presented method. Furthermore, the experimental verification of viability is discussed. The developed framework allows for a high degree of automation, requiring only minimal user input.

## 2. Methods and Derivation

This section serves as the formal analysis of the process requirements and the derivation of the approach. Its structure follows the levels of abstraction, starting with the general goal and motivating decisions accordingly. The aim of the presented method is to enable the manufacture of parts with minimal weight while maximizing stiffness and strength. These requirements directly motivate the choice of material and the use of numerical analysis. As a material, continuous carbon fiber filament has been chosen for its superior performance over chopped fiber filaments and classical filaments used with FDM. This dictates the monolithic design, meaning as few fiber discontinuities as possible. Numerical analysis enables the definition of load cases and boundary conditions to obtain information about the stress distribution, which is key in enabling a load-oriented design. As stated in Section 1.1, FEA and possibly topology optimization are the prevalent choices in this regard, leading to step 0 from Section 1.2. In conjunction with the material requirement of continuity, the principle of reinforcement is applied, which implies the use of the vector fields of principal directions. Their divergent and turbulent properties necessitate optimization methods, as they eliminate the possibility of exactly following the principle of reinforcement. These properties furthermore motivate the nonplanar approach through their out-of-plane characteristic. The field of nonplanar slicing is presented in Section 1.1, isolating two types of nontrivial and nonmanual approaches: decomposition and field-based approaches. As decomposition-based approaches go against the principle of monolithic design, a field-based approach is chosen, where isosurfaces of a scalar field form the constituting layers. Hereby, the choice of a geodesic field would be the only option to ensure a constant layer height. As this severely limits the ability to follow a load-oriented approach, this requirement has to be loosened, itself formulating a requirement of the process. To specify further, the approach presented in this paper is designed for coextrusion or in situ impregnation. Although it can be adapted to also enable dual extrusion, simple towpreg extrusion cannot be utilized, as the layer height cannot be varied due to the fixed ratio of fiber to matrix material. The choice of field is thus open to having an optimized form. This leads to steps 1–3 presented in Section 1.2.

An implication of this is approach via nonplanar layers is the necessity for support material. This stems from the fact that the print bed is assumed to be planar, resulting in a need for transitioning from the bottom planar layer to the first nonplanar layer, which is achieved by adding a buffer of support material below the part geometry. Furthermore, the constituting surfaces can have arbitrary orientation, requiring the identification of an optimal part orientation to minimize the possibility of collisions during manufacturing. As the presented method is aimed at applicability to complex geometries that can result from topology optimization, the support material should be removable even for parts with a high degree of undercut features. This motivates the choice of a water-soluble material. Consequently, an additional extruder is required in the process to enable the printing of support.

With step 4 from Section 1.2 described, pathplanning in the computed part and support surfaces is executed, which has a profound impact on the mechanical performance of the part. This step has no dependencies on the method of slicing if generality is maintained, which was retained in the implementation. This enables the use of any algorithm with this system. In this presented work, great emphasis was put on the minimization of discontinuities to follow monolithic design and to reduce the amount of necessary cutting procedures, relieving strain on the process reliability, as many cutting mechanisms need further optimization. This motivated the choice for a fully continuous approach, which immediately led to the FISO method. As discussed for planar surfaces in [30] and for arbitrary manifold surfaces in [26], it guarantees continuity while introducing low curvature, starting in the maxima of the geodesic distance transform from the boundary and spiraling outwards. Further evaluation should investigate the effects of the choice of the pathplanning algorithm on mechanical performance. With the user specification of travel height, the constructed subpaths for all layers can be connected with travel motions to a contiguous set of machine instructions, which are post-processed to conclude step 5 and 6 from Section 1.2.

The developed framework and software were implemented in Python. For the mathematical aspects of this work, numpy [31] and scipy [32] saw a lot of use in conjunction with the VTK [33] wrapper pyvista [34] for the work with simplicial complexes. Distance computation was performed by use of potpourri3d [35], and networkx [36] was used for computations regarding graphs.

## 3. Algorithm

This section aims to describe the developed algorithm. The subsections correspond to the necessary steps presented in Section 1.2. Input to the algorithm is the tetrahedral mesh *T* with the stress tensor resulting from the FEA attributed to every tetrahedral element *s*. After principal axis transform, the principal stresses and directions can be attributed to *s* as $\sigma_{1,2,3}\left(s\right)$ and $e_{max,med,min}\left(s\right)$, respectively. To minimize the effect of weak interlayer adhesion and increase alignment with the computed principal directions, slices are oriented to have their normals align with the minimal principal direction. To enable fabrication, an optimal part orientation is computed, compatible support structure is generated, and post-processing steps are taken to yield the machine instruction.

### 3.1. Critical Regions

As discussed in Section 1.2, the complex structure of the vector field creates the need for selective incorporation of the input. This is due to the turbulent and divergent structure, which would result in surfaces with high curvature that cannot be used as constituting layers in the 3D-printing process, as printhead collisions and incompatible printing directions would be unavoidable. The approach followed here includes a user parameter k∈(0,1) that denotes the fraction of selective incorporation with 0 representing the full vector field being used and 1 corresponding to no vector input data being considered during computation. Initially, a binary distinction between the critical set of tetrahedral elements $T^{*}$ and the noncritial tetrahedral elements $T^{\circ }$ is made. This is formalized by

(1) $$T^{*}=\left\{s\;|\;\sigma_{1}\left(s\right)>k\cdot \sigma_{max}\right\},\;\sigma_{max}=\max_{s\in T}\left(\sigma_{1}\left(s\right)\right).$$

**Figure 2:**
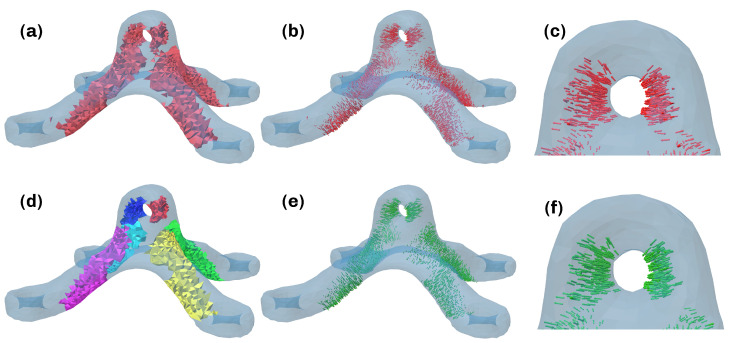
Procedure for computing critical Regions. (**a**) Critical set according to Equation (1). (**b**) Minimum principal directions in critical set. (**c**) Closeup of minimum principal directions. (**d**) Partitioned regions $T_{l}^{*}$. (**e**) Reoriented minimum principal directions in critical regions after flooding. (**f**) Closeup of minimum principal directions in one region after flooding. Vector color green: before reorientation and smoothing, red: after reorientation and smoothing.

This leads to every element whose assigned maximum principal stress exceeds the *k* portion of the highest maximum principal stress in the part being regarded as critical for the performance of the part. An exemplary result of this partition can be found in Figure 2a with the corresponding minimum principal directions displayed in Figure 2b,c. This is followed by a partitioning of $T^{*}$ into the labeled regions $T_{l}^{*}$. This is achieved through a flooding algorithm, which also ensures directional compatibility and alignment within a region. Pseudocode for the algorithm can be found in the Supplementary Material. The noncritical region is denoted as $T^{\circ }:=T_{0}^{*}=T\setminus T^{*}$ with index 0. While iterating over all cells with current cell *i*, the flooding algorithm maintains a list of wavefront cells *I* and candidates *C* that is computed from the first- and second-degree neighbors within the critical set by excluding the wavefront *I*, the already assigned indices *R*, and the already considered indices *F*. Every candidate is tested for its alignment with the current element. If the absolute value of the dot product exceeds the user-defined value α, it is added to the wavefront *I*. If the dot product is negative, the direction of the right-handed system of principal directions is reversed to align it with the current element’s system. After all critical tetrahedra are examined and have been assigned labels, the iteration terminates, yielding the regions $T_{l}^{*}$. An example of the isolated regions with the rejected vectors removed can be seen in Figure 2d. The corresponding aligned minimum principal directions are displayed in Figure 2e,f.

### 3.2. Vector Field

Having determined the critical regions, ensuring inner alignment among the direction vectors, the next step is to compute the vector field in the remaining part. A key part in this computation is the extrapolation of the vector field via minimization of the Dirichlet energy, which expresses a function’s deviation from a constant. This energy is given by

(2) $$E_{D}\left(u\right):=\int_{\Omega }u\Delta u\;dA,$$

for a function **u**. This leads to a notion of smoothness when minimizing the energy. To achieve this with set boundary conditions, the Laplace problem

(3) $$\begin{array}{cc} \Delta u=0\; & \;\mathrm{on}\;\Omega \\ u=g\; & \;\mathrm{on}\;\partial \Omega \end{array}$$

is solved. Functions u that satisfy these conditions are called harmonic and have a variety of helpful properties. To solve this problem on a tetrahedral mesh, the Laplace operator’s discrete solution is applied by solving a linear system. This is achieved by utilizing the dual complex of the simplicial complex T. In the following, $s_{f_{i}}^{\left(l\right)}$ and $s_{f_{i}}^{\left(r\right)}$ will denote the left and right tetrahedrons containing the face $f_{i}$ respectively. The set $F^{\circ }\left(T\right)=\left\{f_{i}\in F\left(T\right)\;|\;\neg \left(s_{f_{i}}^{\left(l\right)}\in T^{*}\wedge s_{f_{i}}^{\left(r\right)}\in T^{*}\right)\right\}$ denotes the set of inner faces, which do not have both their associated tetrahedra lie inside a critical region. These faces have at least one tetrahedron located inside the set $T^{\circ }=T\setminus T^{*}$. $A_{f_{i}}$ is used to measure the area of a face $f_{i}\in F\left(T\right)$. With $F^{\circ }\left(T\right)$ we can define the sets of left and right boundary faces $B_{l}^{*}=\left\{f_{i}\in F^{\circ }\;|\;s_{f_{i}}^{\left(l\right)}\in T^{*}\right\}$ und $B_{r}^{*}=\left\{f_{i}\in F^{\circ }\;|\;s_{f_{i}}^{\left(r\right)}\in T^{*}\right\}$ of the critical regions. These sets form the discrete equivalent to the boundary ∂Ω from Equation (3), on which the boundary conditions are defined. The set $T^{\circ }$ is the equivalent to Ω from Equation (3). With these definitions, the energy

(4) $$E_{V}=\sum_{f_{i}\in F^{\circ }\left(T\right)}A_{f_{i}}{∥v\left(s_{f_{i}}^{\left(l\right)}\right)-v\left(s_{f_{i}}^{\left(r\right)}\right)∥}^{2}$$

with the boundary conditions

(5) $$\begin{array}{cc} \Delta v(s)=0,\; & \forall s\in T^{\circ }\\ v\left(s\right)=\rho \cdot e_{min}\left(s\right),\; & \forall s\in T^{*} \end{array}$$

can be established on the mesh T, with ρ∈{−1,1} incorporating the ambiguity of the right-handed systems resulting from the principal axis transform. This system is solved efficiently in block form to yield the desired vector field in $T^{\circ }$.

**Figure 3:**
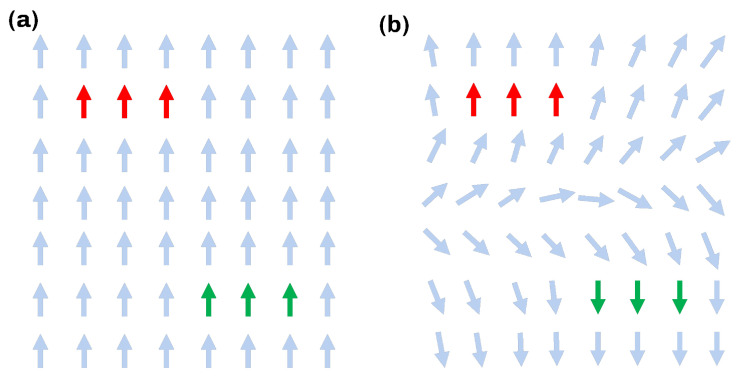
Representation of the effect of orientation incompatabilities of the critical regions. (**a**) Harmonic orientation. (**b**) Disharmonic orientation.

If the process described above were to be applied directly to the result from Section 3.1, the turbulent and divergent vectors in the critical regions and their global orientation ambiguity, which is expressed by ρ in (5), would lead to poor manufacturability, due to the high curvature of the generated surfaces. The first step to mitigate this is to smooth the vector field $E_{min}\left(x\right)$ inside the critical regions by iteratively applying the Laplacian $n_{s}$ times. To remove their global orientation ambiguity, the harmonic variation of the gradient is further minimized by testing for the optimal orientation of all critical regions. The necessity for such an optimization is illustrated in Figure 3. In this work, it was achieved by computing an energy over T that measures the variation in orientation of neighboring vectors. This energy is denoted by

(6) $$E_{W}=\sum_{f_{i}\in F^{\circ }\left(T\right)}A_{f_{i}}{\left(1-v\left(s_{f_{i}}^{\left(l\right)}\right)\cdot v\left(s_{f_{i}}^{\left(r\right)}\right)\right)}^{2}.$$

To find an optimal orientation for all critical regions, the energy is computed for all possible permutations, and the configuration with the lowest value is chosen. The pseudocode for this procedure can be found in the Supplementary Material. With this method, the orientational compatability of the critical regions can be greatly increased, and most turbulences of *V* are removed.

### 3.3. Optimized Scalar Field

With the vector field computation finished in the previous step, the constituting layers have to be determined. This is done by computing a scalar field and extracting the layers as isosurfaces of this field. The first step is achieved by minimizing the difference of the scalar field’s gradient and the vector field. Problems of this structure are known as Poisson problems, i.e., when Δu≠0 on Ω in Equation (3). A physical interpretation is the search of a potential field, which best explains the given vector field. The problem is formalized by the energy minimization

(7) $$\underset{G}{min}\int_{M}{\left|\nabla G-V\right|}^{2}$$

If *V* actually describes a gradient field, the solution will be exact. To solve this problem computationally, it is discretized and applied to the nodes and cells of the mesh, resulting in

(8) $$G\left(s\right)=\underset{G}{argmin}\sum_{s\in T}w_{i}{\left|\nabla G\left(s\right)-V\left(s\right)\right|}^{2},$$

with the weights $w_{i}$ being able to increase the impact of specific regions, which is only used in the computation of the support structure. The solution is again computed efficiently in block form. After acquiring the values of the scalar field on the nodes via the interpolation functions $\phi_{i}$ on the mesh, it is normalized and inverted by applying

$$g_{norm}=1-\frac{g-min(g)}{max(g)-min(g)}.$$

**Figure 4:**
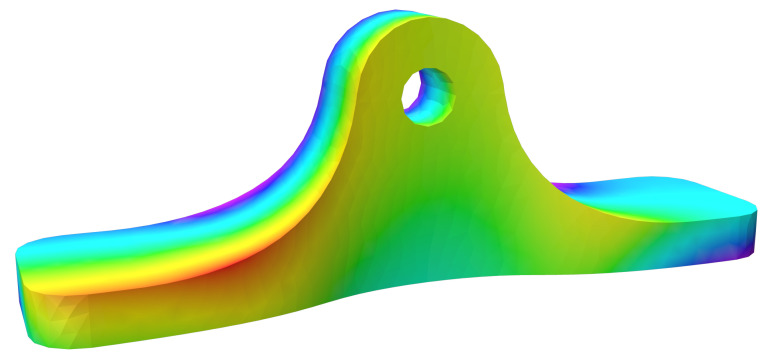
Calculated normalized scalar field, values indicated by color ∈ [0, 1].

An exemplary result of this computation can be found in Figure 4.

### 3.4. Slicing and Support

The process of slicing and generating support is presented in the following. The three subsections correspond to the different classes of computation steps.

#### 3.4.1. Slicing

**Figure 5:**
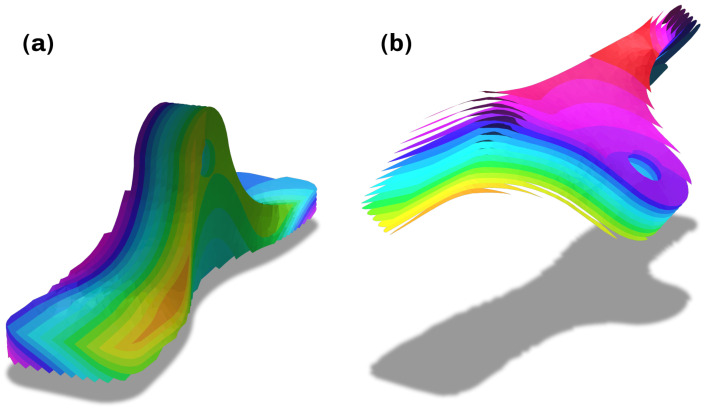
(**a**) Constituting surfaces. (**b**) Reoriented slices. Original scalar field values indicated by color ∈ [0, 1], $h_{s}=0.5$ mm.

To ensure high compliance with the desired layer height, the extraction of the constituting surfaces is performed in two steps. The scalar field’s range is scaled to a range of [0,100] and sliced with a step size of 1. As a next step, the mean of the average distances over all surface points is determined. With this information, the step size $l_{step}$ is computed by simple division, now corresponding to the layer height $h_{s}$ set by the user. Obtaining the isosurfaces is done by applying the contour filter from pyvista [34], which uses the interpolation functions to generate new polygons depending on the scalar values at the vertices. The nonplanar nature of the surfaces is shown clearly in the exemplary result in Figure 5b.

#### 3.4.2. Orientation and Preprocessing

The orientation of the workpiece has a profound impact on manufacturability, production time and support geometry. Without reorientation measures, a collision of the printhead and printbed is nearly unavoidable. The optimal orientation is computed by averaging the mean of all surface normals in every cell over all layers. The relevant reorientation is then given by a rotation of this vector into the positive z-direction. By iterative translation of these cells, a distance $h_{b}$ is created between the surfaces and the printbed, which results from the number of buffer layers given by the user parameter $n_{buff}$. In Figure 5, the reoriented slices can be seen in (b).

#### 3.4.3. Support

In order to be able to print the part while starting from a flat printbed, support material is necessary. This allows for the transition from the first planar layer on the printbed to the first nonplanar layer of the part. To compute the geometry, all cells in need of support are determined by checking if their normal has a negative component in the z-direction. The vertices of the identified cells are iteratively translated down to result in a pointcloud, which forms the base of the support geometry. A hull is created by extruding the part’s shadow. This is used to clip the geometry defined by the pointcloud. Clipping by the part’s outer surface results in the support geometry S.

**Figure 6:**
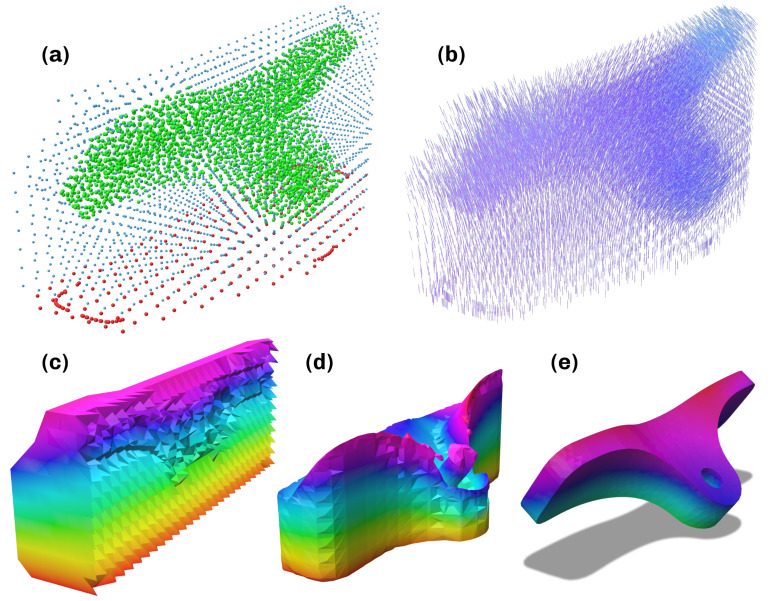
Computation of the scalar field in the convex hull. (**a**) Pointcloud with points marked as green: part (T), red: printbed (D) and blue: (H∖(T∪D)). (**b**) Extrapolated vector field. (**c**) Scalar field in convex hull. (**d**) Scalar field in support. (**e**) Scalar field in convex hull, values indicated by color ∈ [0, 1].

The next step in the pipeline concerns the construction of constituting layers for the support material. A major factor during computation is the compatibility of support and part surfaces. If the support were to be built up by planar layers, an increase in material changes and high risk of obstruction by already printed layers would be highly likely. To remove these complications, the support layers are computed with the same procedure used for slicing the part, with the added requirement of the first layers having to be planar, as they are in direct contact with the printbed. To this end, the scalar field has to be extrapolated to the support geometry, based on the requirements of planar initial layers and compliance with the constituting surfaces in the part. For the extrapolation, a convex hull H is computed from the combined vertices of the part and the support meshes seen in Figure 6a. In a process similar to the approach in Section 3.2 and Section 3.3, the discrete Dirichlet energy is minimized. To ensure the requirement of planarity near the printbed, a region $D=\{s\in H\;|\;\exists p_{i}\in s,\;\langle p_{i},{\left(0,0,1\right)}^{T}\rangle <\frac{h_{b}}{2}\}$ is taken into account, which is shown in red in Figure 6a. The boundary conditions from (4) are given by

(9) $$\begin{array}{cc} \Delta v_{\mathrm{neu}}\left(s\right)=0,\; & \forall s\in H\setminus T\\ v_{\mathrm{new}}\left(s\right)=v\left(s\right),\; & \forall s\in T\\ v_{\mathrm{new}}\left(s\right)={\left(0,0,1\right)}^{T},\; & \forall s\in D \end{array}$$

to ensure the stated requirements. Solving the minimization is performed in following the steps in Section 3.2, which results in the vector field shown in Figure 6b and Section 3.3, which results in the scalar field in the convex hull shown in Figure 6c. During this computation, the weights $w_{i}$ in the region D are set with a value w>1 to further prioritize planarity. Finally, the scalar field on the respective meshes can be extracted by clipping with the corresponding geometries, seen in Figure 3d,e.

With the optimized scalar fields in part and support, the slices for both can be obtained with the same procedure as described in Section 3.4.1, resulting in compatible surfaces. What have not been examined at this point are the actual interlayer distances resulting from this process. As an optimization is performed, the computed layers have to be checked for lying outside of the user-defined minimum and maximum layer distances $\left[d_{min},d_{max}\right]$, and intermediate layers have to be inserted where necessary. This is achieved by raytracing from every vertex of a surface along its normal. The distances are determined and candidate vertices are identified as the midpoints, for which the distance exceeds $2\cdot d_{min}$. This also informs the requirement of $2\cdot d_{min}<d_{max}$ to ensure the possibility of generating intermediate layers. The average scalar value of the candidate point is then utilized to extract a new isosurface from the scalar field. The resulting collection of constituting surfaces forms the base on which pathplanning can be executed.

### 3.5. Pathplanning

In this work, the first-in-spiral-out algorithm from [30] was chosen, as the guaranteed continuity was prioritized due to the usage of continuous carbon fiber filament. This choice is in no way final, as any nonplanar pathplanning algorithm can be chosen, and more adapted pathplanning algorithms should be a topic of further research. The only user input to the algorithm is the path width $w_{p}$. Before applying the algorithm to every surface, they are preprocessed to prevent degenerate or incorrect paths in geometrical edge cases. Surfaces with a maximal geodesic distance from their boundary smaller than $\frac{1}{2}w_{p}$ are discarded, as not a single path can be placed upon them at any point. Next, a procedure similar to the opening operator from image morphology is applied to separate surfaces with thin connecting sections. To this end, the surfaces are clipped at a value $t_{o}$ of the geodesic distance field from their boundary and expanded again by the same value. This removes thin and sharp structures from the surface and separates surfaces at regions that are thinner than $2t_{o}$. The process is executed for both $t_{o}=\frac{1}{2}w_{p}$ and $t_{o}=\frac{3}{4}w_{p}$. In the nominal case, $t_{o}=\frac{1}{2}w_{p}$ is chosen. Only when $t_{o}=\frac{3}{4}w_{p}$ results in a multiplicity of surfaces and $t_{o}=\frac{1}{2}w_{p}$ does not does the algorithm proceed with the newly separated surfaces corresponding to $t_{o}=\frac{3}{4}w_{p}$ to prevent numerical errors.

**Figure 7:**
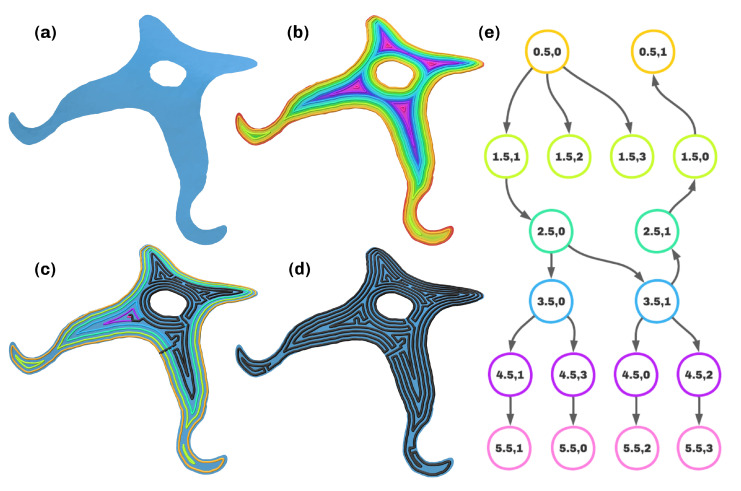
Overview of the FISO algorithm. (**a**) Freeform surface. (**b**) Geodesic distance field with isocontours. (**c**) Rerouting process and path. (**d**) Globally continuous path. (**e**) Minimal spanning tree on the contour graph.

By iterating over the isovalues, pathplanning is executed for every surface. As this procedure of routing is explained in detail in [26,30], it will be given in short form in this work. The process is depicted in Figure 7. After obtaining the isovalues as lying in $\left[\frac{1}{2}w_{p},max\left(g\right)-\frac{1}{2}w_{p}\right]$ with a step size of $w_{p}$, the contour filter from pyvista [34] is applied again, to yield the isocurves of the geodesic field from the boundary. After some post-processing and resampling of the isocurves, the digraph of contours G is created. G contains a node for every contour and edges between contours of neighboring isovalues, whose weight corresponds to their minimal distance and whose direction corresponds to increasing distance from the boundary. To find the order of rerouting, a minimum spanning tree T is calculated from G. Rerouting is the procedure of connecting the isocontours of a surface to a continuous path. The resulting path has the property of always rising into the maxima of the geodesic distance field, then spiraling out with switching directions. This approach results in low curvature paths, as the long segments exhibit gradual direction changes, especially when traveling to a geodesic maximum. Rerouting is done recursively, with the biggest challenges being the construction of connecting segments between isocontours and incorporating the turning direction of the curves. Pseudocode for the very briefly described procedures can be found in the Supplementary Material. After recursively traversing the whole tree T and connecting the isocontours at the optimal points, the continuous path is returned. If it exceeds the minimum length, typically given by the distance of the printing nozzle to the fiber cutting apparatus, the rates of extrusion are calculated. They correspond to the layer height in every point, meaning the actual distance to the layer below, which has to be computed carefully due to the presence of intermediate layers.

The final steps of pathplanning include the interlaminar connection and collision detection. To connect the continuous paths, travel motions with a height of $l_{tr}$ are inserted along the point normals of start and end point. Collision detection is done individually for every layer, maintaining an object that contains the already printed geometry. This is used to check for every point if a collision with a simplified printhead geometry is detected. Finishing this procedure concludes the pathplanning process.

### 3.6. Post-Processing

As the subpaths were created with a uniformly high resolution, the final path contains many collinear segments. These have to be removed in order to keep the machine instruction count and memory requirement to a minimum. The parameters $t_{p}$ and $t_{n}$ correspond to the minimal scalar product value of the two vectors connecting three neighboring points and the normal vectors of two path points, respectively. In an iteration over all path points, the points that have a deviation smaller than those given by the parameters are removed, and the path is reconnected by a linear segment. The last step in the pipeline is the post-processing of the path to machine instructions. This is done by a separate module that is specific to the machine used.

## 4. Results

**Figure 8:**
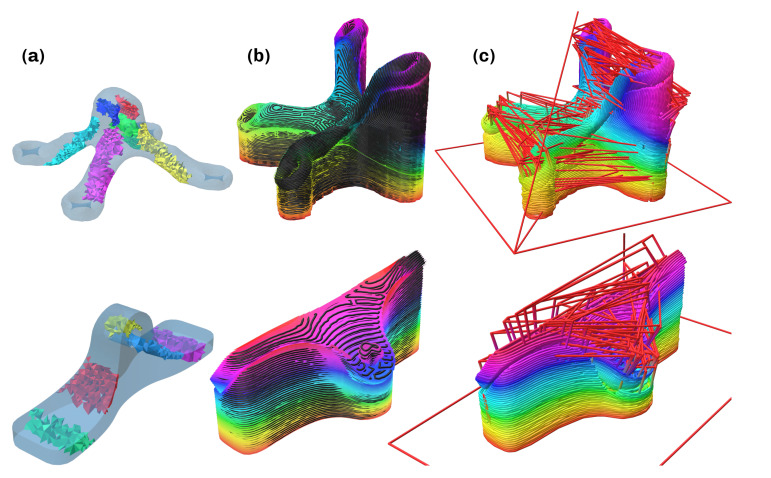
Results of computation for part A (**top**) and B (**bottom**). (**a**) Critical regions. (**b**) Layers and subpaths. (**c**) Final path with travel motions in red.

In this section, the conducted verification of viability is documented. Two parts, which can be seen in Figure 8a, were analyzed with the developed method of which one was chosen to execute the initial testing of the procedure. Both parts represent variants of mounting brackets, and the load was assumed to point in a positive z-direction at the upper mounting hole in both cases. Part A was constructed and analyzed using topology optimization in the software Abaqus [37], while part B was directly constructed and analyzed in the open source CAD software FreeCAD [38]. The definition of load cases has a high impact on the computational results and defines the mechanical performance in the evaluation. Part properties and the chosen user-defined parameters can be found in Table 1.

Computation was done on a Intel^®^ Core™i7-1165G7 CPU (8 Cores @ 2.8GHz) + 16GB RAM running Manjaro Linux. The execution times are noted in Table 2. For the higher resolution part A, the total time was 23 min, for part B with lower resolution, 5.5 min. Pathplanning for every surface was identified as the bottleneck, but the number of critical regions has an high impact as well, as the computation of optimal direction scales with $2^{n}$.

**Figure 9:**
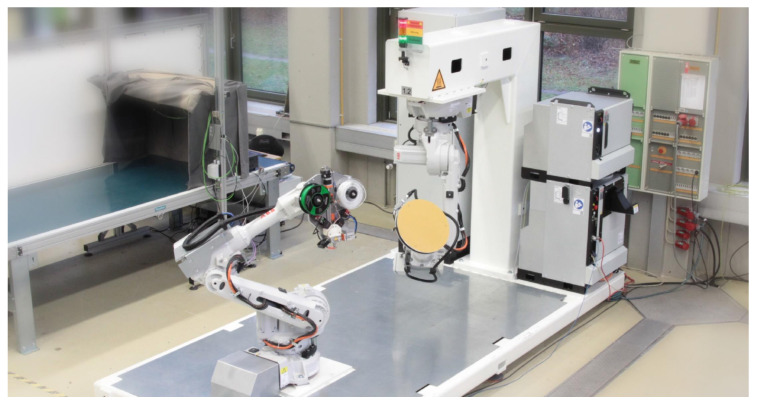
Experimental setup and robotic 3D-printing machine used in the verification of viability [39].

**Figure 10:**
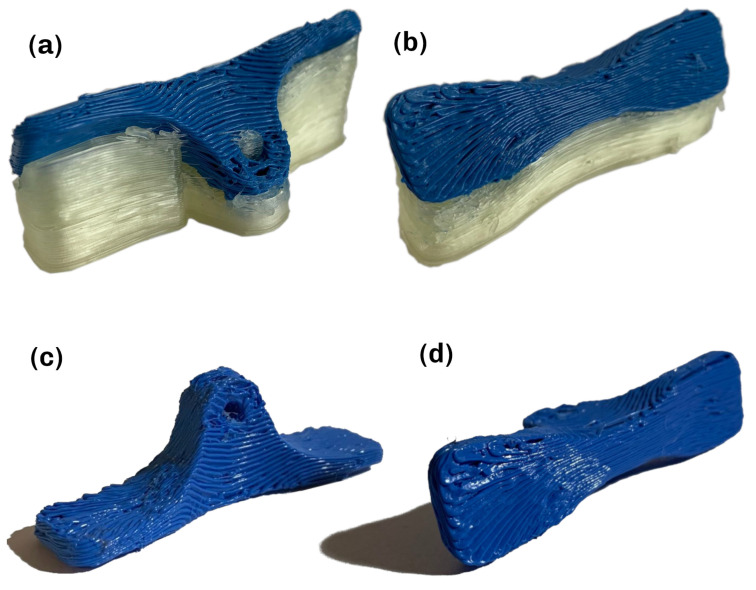
Results of the final test. (**a**) Part with support material (front). (**b**) Part with support material (back). (**c**) Part without support material (front). (**d**) Part without support material (bottom).

The experimental verification of viability was done on a robotic 3D-printing machine seen in Figure 9, which is presented and evaluated in [39]. This system is currently not capable of printing with varying layer height, as it is not equipped with a coextrusion printhead. This represents the major limiting factor of the experimental evaluation, as no carbon fiber filament could be used for the an experimental evaluation at this point. Hence, all experiments were conducted with a classical PLA filament as a stand-in until a coextrusion printhead becomes available. PVA filament was used as the material for the support structure for its water-soluble characteristics. Part B was chosen for the experimental evaluation to ensure comparability and enable many iterations in a short time, as it has a decreased print time. Pictures with explanations of these iteration milestones can be found in the Supplementary Material. After simulating the robot trajectory and checking for collision or singularities, the printing process was started. The initial tests were performed without material changes, building up the part with only PLA. While implementing the multi-material approach and fixing relative errors, determining the material extrusion factor was an important step, as nozzle movement, extrusion and layer height have to be matched to ensure correct material deposition. Problems due to oozing that could not be fixed by adapting the retraction were removed by including a wiping apparatus, which was used to clean the nozzle after every material change. Figure 10a,b show the results of the final test with the PVA support material. The total time for printing was 134 min, and removing the support material by submerging the part in water took 20 h until the material was fully dissolved. The final part is shown in Figure 10c,d.

When comparing the resulting part from Figure 10 to the expected geometry of the part and surface from Figure 5, the general layer structure can be validated to have been achieved by the printing process. Defects are visible in Figure 10a, especially in the area around the mounting hole, due to the presence of shorter segments. Further improvements of the printing parameters and setup are expected to increase material quality in this regard. Other defects can be attributed to the comparatively large nozzle diameter and layer heights, which were used to decrease manufacturing time. Better results should be achievable by reducing the nozzle diameter, layer heights or printing speeds. Furthermore, the effect of factors such as over- and underextrusion, printing temperature and the influence of the hotend and extruder choices should be investigated. In conclusion, it can be said that the developed pipeline results in executable machine instruction that produces parts matching the desired geometry and structure.

## 5. Conclusions and Prospects

A method for load-oriented nonplanar slicing and pathplanning is presented in this work, which is specially designed for the manufacture of continuous carbon fiber parts with the process of FDM. This represents a major step in the development of CFRP adapted slicing techniques for additive manufacturing, which is one of the major bottlenecks in the field. The combination of load adequacy and the continuous carbon fiber material enables the possibility of manufacturing parts with a high strength to weight ratio, while taking full advantage of the benefits of AM. To ensure full coverage of the fabrication preparation process, the pipeline was developed and presented from start to finish, taking the load analysis as input and directly outputting machine instructions. This sequence of computations was presented in detail, evaluated and contextualized when necessary. Moreover, the derivation from the process requirements and research goal was conducted and documented. To yield nonplanar slices, the concept of isosurfaces of an optimized scalar field was employed. This makes the use of load analyses result in load-oriented parts, as the direction of the weaker interlayer adhesion is oriented along the direction of minimum principal stress, furthermore ensuring that the fiber directions lie in the plane of higher stress, which is spanned by the maximum and medium principal stress directions. The conceptually decoupled pathplanning was chosen and implemented to utilize the FISO algorithm, guaranteeing complete continuity within every constituting surface, respectively. This eliminates the need for cutting the fiber during the printing process of a layer. Finally, the developed method was applied to exemplary parts and evaluated regarding computational cost. The construction of the part geometries and finite-element-analysis was performed by applying both topology optimization and classical computer-aided design, demonstrating the possibility for using proprietary and open-source software with the developed method. A verification of viability was conducted by generating machine instruction for an exemplary part and executing a series of tests on a multi-axis printer, showing the capability and adequacy of the approach. The produced parts matched the desired geometry and structure resulting from the computation.

Further work includes the load case analysis with a focus on generating meaningful input data, as this method is highly dependent on the definition of load cases. Additionally, a focus should be the investigation of further adaption of the scalar field generation and especially the pathplanning methods in conjunction with the development of a capable printing head and the principle of monolithic design. With the indispensable coextrusion technology, which enables the usage of a carbon fiber filament while being able to vary layer height, the next step is the incorporation of continuous carbon fiber material into the evaluation and extensive parameter studies. Tensile tests should be carried out to further validate the effectiveness of the approach.

This paper precedes, to the best knowledge of the authors, any load-oriented nonplanar slicing and pathplanning method for the additive manufacture of volumetric continuous carbon fiber parts and represents a major step in achieving the goal of fabricating complex parts with this promising material and flexible process.

## Figures and Tables

**Table 1:** Part properties and user-defined parameters used for verification of viability.

Part	Dim. [mm]	# Tets	k	α	$n_{s}$	$h_{s}$	$n_{\mathrm{buff}}$	$\left[d_{\min},d_{\max}\right]$	$w_{p}$	$l_{tr}$
A	52 × 52 × 27	32,666	0.16	0.75	200	0.35	15	[0.2;0.5]	0.9	5
B	80 × 20 × 28	8514	0.46	0.75	200	0.35	10	[0.2;0.5]	0.9	5

All values rounded to two decimal places

**Table 2:** Computation times for the individual pipeline steps in seconds.

Part	$T^{*}$	V(·)	G(·)	Slices	Ori.	S	Paths	Path	Post
A	12.86	71.53	18.12	15.66	0.97	126.52	892.88	254.55	38
B	4.82	8.71	3.52	2.74	0.07	34.11	204.58	62.55	8.44

All values rounded to two decimal places

## Data Availability

Pseudocode and verification of viability documentation in Supplementary Material.

## Supplementary Materials

The following supporting information can be downloaded at: https://www.mdpi.com/article/10.3390/ma16030998/s1, Figure S1: Results of the first test series. After first layers of the part geometry, visible in (b) in red the process was terminates due to defects; Figure S2: Results of the second test series. Relative error in coordinate frames and collision due to part orientation; Figure S3: Results of the third test series. Problems due to oozing, stringing and material extrusion; Figure S4: Results of the fourth test series. Manufacturing process could be completed and the support material was successfully dissolved; Pseudocode S1: Region Flooding; Pseudocode S2: Orientation and Extrapolation; Pseudocode S3: Recursive rerouting; Pseudocode S4: Computation of the connecting segments; Pseudocode S5: Connection of the path to one isocontour.

## Abbreviations

The following abbreviations are used in this manuscript:

AM	Additive manufacturing
CAD	Computer-aided design
CFRP	Carbon-fiber-reinforced polymers
FDM	Fused deposition modeling
FEA	Finite-element-analysis
FEM	Finite-element-methods
FISO	First in spiral out
PLA	Polylactic acid
PVA	Polyvinyl alcohol
SIMP	Solid isotropic material with penalization
SOMP	Solid orthotropic material with penalization
