# Supplementary material for: Load-Oriented Nonplanar Additive Manufacturing Method for Optimized Continuous Carbon Fiber Parts
Source: Materials (Basel). 2023 Jan 21;16(3):998. doi: 10.3390/ma16030998 (PMC9920591; doi:10.3390/ma16030998)
Supplement: Supplementary file 1 [file materials-16-00998-s001.zip › materials-2113945-supplementary.pdf]

Supplementary Material for
Load-Oriented Nonplanar Additive Manufacturing Method for Optimized Continuous Carbon Fiber Parts

1 Pictures of Verification of Viability

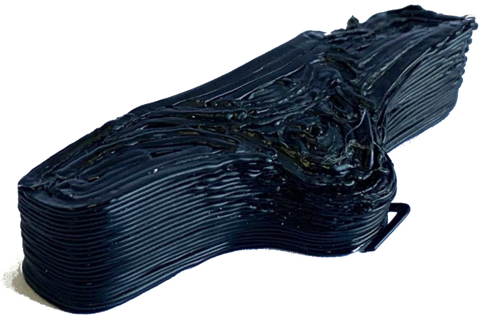

(a) No material change, PLA.

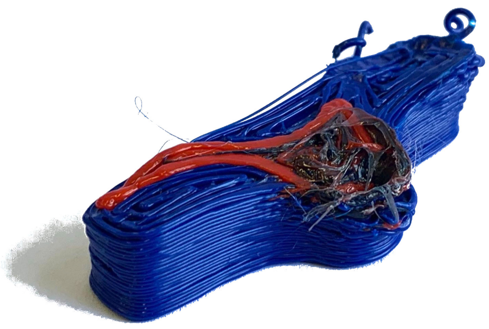

(b) With material change, multiple colors - PLA.

Figure S1: Results of the first test series. After first layers of the part geometry, visible in (b) in red the process was terminates due to defects.

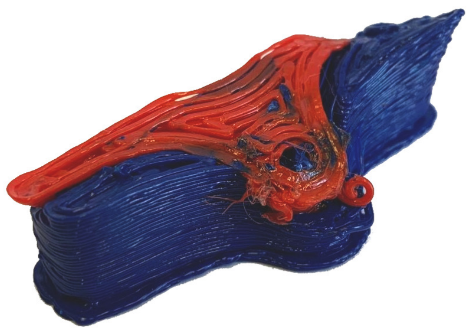

(a) Relativfehler der Werkzeugkoordinatensysteme.

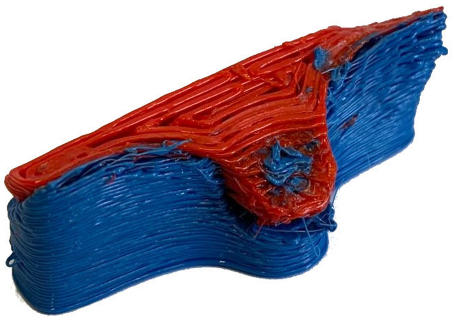

(b) Abbruch vor Kollision.

Figure S2: Results of the second test series. Relative error in coordinate frames and collision due to part orientation.

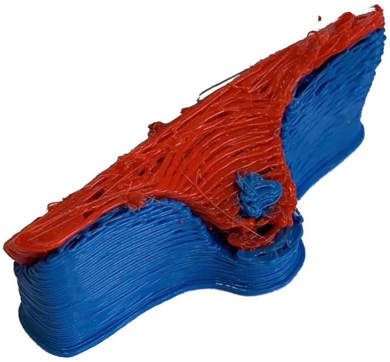

(a) Fertigungsergebnis aus PLA.

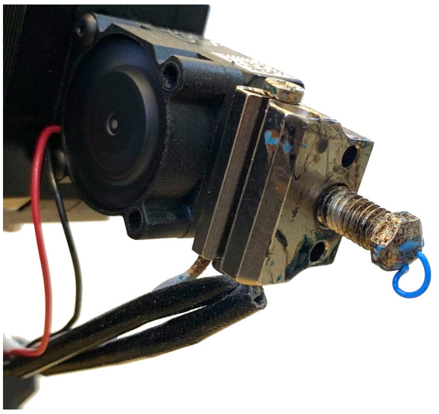

(b) Düse mit Materialüberschuss.

Figure S3: Results of the third test series. Problems due to oozing, stringing and material extrusion.

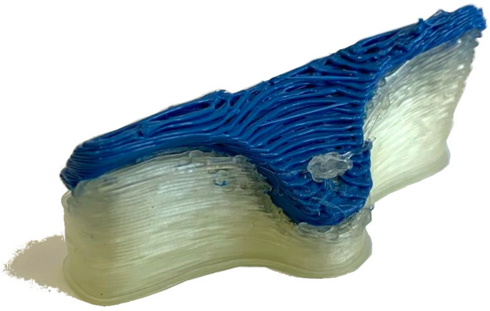

(a) Gedrucktes Bauteil aus PLA mit Stützstruktur aus PVA.

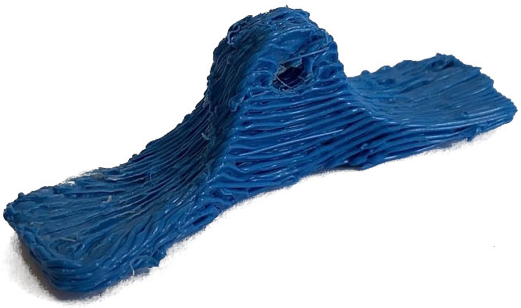

(b) Gedrucktes Bauteil, Versuchsergebnis.

Figure S4: Results of the fourth test series. Manufacturing process could be completed and the support material was successfully dissolved.

2 Pseudocode

2.1 Region Flooding

Used notation: sets I for current indices, R for elements in the current region, F for the elements for which calculation is finished and C for the candidates.

Algorithm S1 Region Flooding

Require: $\alpha \in [0,1],\ n_{region} \in \mathbb{N}^{+}$
  $l \leftarrow 1$
  $I \leftarrow \{\ \text{pop}(\mathcal{T}^*)\ \}$
  $R \leftarrow \emptyset$
  $F \leftarrow \mathcal{T} \setminus \mathcal{T}^*$
  while $F \neq \mathcal{T}$ **do**
    if $I \neq \emptyset$ **then**
      $i \leftarrow \text{pop}(I)$
    else if $|R| > n_{region}$ **then**
      reorder(R)
      $\mathcal{T}_l^* \leftarrow R$
      $l \leftarrow l + 1$
      $F \leftarrow F \cup R$
      $I \leftarrow \{\ \text{pop}(\mathcal{T}^* \setminus F)\ \}$
      $R \leftarrow \emptyset$
    end if
    $N \leftarrow \text{neighbors}(i) \cap \text{neighbors}(\text{neighbors}(i))$
    $C \leftarrow (\mathcal{T}^* \cap N) \setminus (I \cup R \cup F)$
    for $c \in C$ **do**
      if $|e_{min}(i) \cdot e_{min}(c)| > \alpha$ **then**
        if $e_{min}(i) \cdot e_{min}(c) < 0$ **then**
          flip_direction(c)
        end if
        $I \leftarrow I \cup \{c\}$
      end if
    end for
    $R \leftarrow R \cup \{i\}$
  end while

2.2 Orientation and Extrapolation

Algorithm S2 Orientation and extrapolation

Require: $\{T_l^*\}_{1,...,n_{regions}}, V$
  $n_{iter} \leftarrow 2^{n_{regions}}$
  $E_{min} \leftarrow \infty$
  $i_{min} \leftarrow 0$
  for $i \in 1, ..., n_{iter}$ **do**
    $o \leftarrow \text{binary}(i)$ ▷ Tupelindex from binary representation
    $V(T_o^*) \leftarrow -V(T_o^*)$ ▷ direction change at tupelindex o
    $V \leftarrow \text{extrapolate}(V)$
    $E_W \leftarrow \text{compute\_energy}(V, T_l^*)$
    if $E_W < E_{min}$ **then**
      $E_{min} \leftarrow E_V$
      $i_{min} \leftarrow i$
    end if
    $V(T_o^*) \leftarrow -V(T_o^*)$ ▷ Change back
  end for
  $o \leftarrow \text{binary}(i_{min})$
  $V(T_o^*) \leftarrow -V(T_o^*)$
  $V \leftarrow \text{extrapolate}(V)$

2.3 Rerouting

Used notation: Minimal spanning tree of contours T; dictionary of isocontours `line_dict`; number of points n_w, that correspond to the path width w_p after resampling; rerouting distance w_r; path width w_p; current contour c_{curr}; starting index i_{start}; binary list of already connected contours `rerouted`; current path `path`. From the outer function the call to `recreroute()` with the root contour, the starting index 0 and a list of zeros as `rerouted`. The root contour is always set as the outer contour, not an hole contour.

Algorithm S3 Recursive rerouting

```
statics ← (T, line_dict, n_w, w_r, w_p)
function RECREROUTE(statics, c_curr, i_start, rerouted, path)
    C ← childs(c_curr)
    {c_k}_{k∈C} ← line_dict[C]
    n_c ← |{c_k}_{k∈C}|
    if (c_curr is leaf) oder (∀k ∈ C : rerouted[k] is True) then
        return (rerouted, path)
    end if
    while Not all c_k in rerouted do
        P_p ← {i:[] for i ∈ 1, ..., n_c}
        P_c ← {i:[] for i ∈ 1, ..., n_c}
        for i_f ∈ 1, ..., len(path) do
            i ← (i_f + i_start) % len(path)                ▷ % is the modulo operator
            p_closest, c_matching ←find_closest_point(path[k])
            if dist(p_closest, path[i]) < w_r then
                P_p[index(c_matching)].append(path[i])
                P_c[index(c_matching)].append(p_closest)
                I_p, _, _, _ ← get_segment_inds(P_p, P_c, path, c_k, n_w)
                if Segment path[I_p] is longer than w_p then
                    s_p, s_c, m_p, m_c, γ ←get_rerouting_segments(P_p, P_c, path, c_k, n_w)
                    i_start, path ←reroute(s_p, s_c, m_p, m_c, γ)
                    break
                end if
            else
                P_p[index(c_matching)] = []
                P_c[index(c_matching)] = []
            end if
        end for
        rerouted, path ← recreroute(statics, index(c_matching), i_start, rerouted, path)
        rerouted[index(c_matching)] ← True
    end while
end function
```

Used notation: `get_rerouting_segments` gets the point candidates P_r and P_c for the path and the child contour, the path `path`, the current contour c_k, the path width w_p and the number of points n_w, that correspond to the path width w_p after resampling.

Algorithm S4 Computation of the connecting segments

function GET_REROUTING_SEGMENTS($P_p, P_c, path, c_k, w_d, n_w$)
  $I_p, I_c, m_p, m_c \leftarrow$ get_segment_inds($P_p, P_c, path, c_k, n_w$)
  $s_p \leftarrow path[I_p]$; $s_c \leftarrow c_k[I_c]$
  $V_p \leftarrow s_p[1:] - s_p[:-1]$; $V_c \leftarrow s_c[1:] - s_c[:-1]$
  $dir_p \leftarrow mean(V_p)$; $dir_c \leftarrow mean(V_c)$
  $\gamma \leftarrow \langle dir_p, dir_c \rangle$
  while Segment s_c is shorter than w_p **do**
    if No kink in s_c **then** Add previous neighbor from c_k to s_c
    end if
    if No kink in s_c **then** Add previous neighbor from c_k to s_c
    else break
    end if
  end while
  return $s_p, s_c, m_p, m_c, \gamma$
end function

function GET_SEGMENT_INDS($P_p, P_c, path, c_k, n_w$)
  $n_{thresh} \leftarrow \frac{n_w}{2}$
  $m_p \leftarrow (\exists n \in path : n < n_{thresh}) \wedge (\exists n \in path : n > len(path) - 1 - n_{thresh})$
  $m_c \leftarrow (\exists n \in c_k : n < n_{thresh}) \wedge (\exists n \in path : n > len(c_k) - 1 - n_{thresh})$
  if $\neg m_p \wedge \neg m_c$ **then** $I_p \leftarrow sort(P_p)$; $I_c \leftarrow sort(P_c)$
  else if $m_p \wedge \neg m_c$ **then** $I_p \leftarrow modulo\_sort(P_p)$; $I_c \leftarrow sort(P_c)$
  else if $\neg m_p \wedge m_c$ **then** $I_p \leftarrow sort(P_p)$; $I_c \leftarrow modulo\_sort(P_c)$
  else if $m_p \wedge m_c$ **then** $I_p \leftarrow modulo\_sort(P_p)$; $I_c \leftarrow modulo\_sort(P_c)$
  end if
  return (I_p, I_c, m_p, m_c)
end function

function MODULO_SORT(P)
  $I_u \leftarrow sort(P)$
  $i_s \leftarrow len(P_p) - 1 - argmin(P_p)$
  $i_e \leftarrow len(P_p) - 1 - argmax(P_p)$
  $s \leftarrow I_u[i_e :] \circ I_u[: i_s + 1]$
  return s
end function

Algorithm S5 Connection of the path to one isocontour

```
function REROUTE(path, c_k, s_p, s_c, m_p, m_c, γ)
    i_p^in ← s_p[0]
    i_p^out ← s_p[−1]
    if γ < 0 then
        i_c^in ← s_c[−1]
        i_c^out ← s_c[0]
    else
        i_c^in ← len(c_k) − 1 − s_c[0]
        i_c^out ← len(c_k) − 1 − s_c[−1]
        c_k ← reverse(c_k)
    end if
    if ¬m_p ∧ ¬m_c then
        path ← path[: i_p^in + 1] ∘ c_k[i_c^in :] ∘ c_k[: i_c^out + 1] ∘ path[i_p^out :]
    else if m_p ∧ m_c then
        path ← path[i_p^in] ∘ c_k[i_c^in : i_c^out + 1] ∘ path[i_p^out : i_p^in + 1]
    else if ¬m_p ∧ m_c then
        path ← path[: i_p^in + 1] ∘ c_k[i_c^in : i_c^out + 1] ∘ path[i_p^out :]
    else if m_p ∧ ¬m_c then
        path ← path[i_p^in] ∘ c_k[i_c^in :] ∘ c_k[: i_c^out + 1] ∘ path[i_p^out : i_p^in + 1]
    end if
end function
```
